# Optically responsive supramolecular polymer glasses

**DOI:** 10.1038/ncomms10995

**Published:** 2016-03-17

**Authors:** Diederik W. R. Balkenende, Christophe A. Monnier, Gina L. Fiore, Christoph Weder

**Affiliations:** 1 Adolphe Merkle Institute, University of Fribourg, Chemin des Verdiers 4, CH-1700 Fribourg, Switzerland

## Abstract

The reversible and dynamic nature of non-covalent interactions between the constituting building blocks renders many supramolecular polymers stimuli-responsive. This was previously exploited to create thermally and optically healable polymers, but it proved challenging to achieve high stiffness and good healability. Here we present a glass-forming supramolecular material that is based on a trifunctional low-molecular-weight monomer ((UPyU)_3_TMP). Carrying three ureido-4-pyrimidinone (UPy) groups, (UPyU)_3_TMP forms a dynamic supramolecular polymer network, whose properties are governed by its cross-linked architecture and the large content of the binding motif. This design promotes the formation of a disordered glass, which, in spite of the low molecular weight of the building block, displays typical polymeric behaviour. The material exhibits a high stiffness and offers excellent coating and adhesive properties. On account of reversible dissociation and the formation of a low-viscosity liquid upon irradiation with ultraviolet light, rapid optical healing as well as (de)bonding on demand is possible.

## Abstract (web summary)

The ability to heal whilst maintaining original functionality is important for establishing materials with superior longevity. Here, the authors demonstrate a supramolecular polymeric coating which exhibits excellent optical and mechanical properties, even following damage repair.

## Graphical abstract

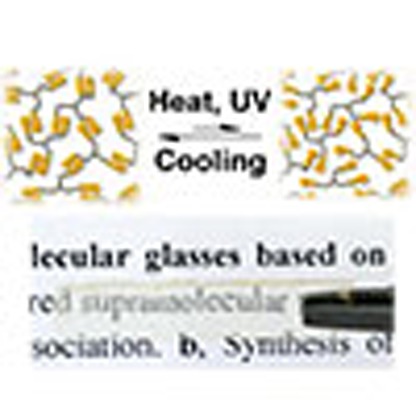

Supramolecular polymers are assembled from monomeric building blocks through non-covalent, directional interactions, such as H-bonding, π–π stacking and ligand–metal complexation^1^. The nature and strength of useful supramolecular interactions vary widely. The reversible and in many cases dynamic nature of supramolecular binding can be used to impart a wide range of stimuli-responsive characteristics^2^,^3^, including mechanochromism^4^,^5^, bioactivity^6^ and mechanical morphing^7^. The possibility to temporarily reduce the molecular weight of the supramolecular assemblies by shifting the equilibrium to the monomer side upon exposure to an appropriate stimulus can also be used to create healable (or self-healing, if the system is sufficiently dynamic under ambient conditions) polymers. This is the result of an increased chain mobility and decreased viscosity upon disassembly, which enable the material to flow and fill cracks and gaps before the original material is reformed by shifting the equilibrium back to the assembled state^8^,^9^,^10^,^11^,^12^. The same approach can be used to create reversible adhesives that permit (de)bonding on demand^13^. Examples of thermally healable materials based on this general concept include hydrogen-bonded rubbers based on telechelic poly(ethylene*-co*-butylene) functionalized with ureido-4-pyrimidinone (UPy) units^8^,^14^, elastomer networks based on fatty acids, ethylene diamine and urea^9^, and phase-separated elastomers based on a polystyrene-polyacrylic acid brush copolymer^10^. Optically healable supramolecular polymers, which are advantageous because the stimulus can be applied in a targeted manner, have also been realized, for example, on the basis of telechelic poly(ethylene-*co*-butylene) that was chain-terminated with terdentate ligands and assembled into a polymer with stoichiometric amounts of Zn^2+^ or Eu^3+^ salts^15^,^16^,^17^,^18^. However, on account of the dynamic nature of the supramolecular motifs employed, and the use of building blocks with low glass transition temperature (*T*_g_), virtually all known healable supramolecular polymers exhibit a low resistance to mechanical stress. This problem can to a certain extent be overcome by the introduction of a reinforcing (nano)filler^19^,^20^,^21^, but even with this improvement the stiffness (storage modulus of <250 MPa) and strength (tensile strength of <5 MPa) are rather low and stifle the exploitation of such materials as a replacement of glassy thermoset resins in coatings, adhesives and other applications^22^. In this study, we show that the combination of high stiffness imparted by supramolecular moieties and stimuli-responsive behaviour is accessible with supramolecular polymer glasses based on a low-molecular-weight, tri-functional supramolecular monomer, which assembles into a disordered supramolecular network. Although *molecular* glasses represent a well-investigated class of materials^23^,^24^,^25^, *supramolecular polymer* glasses based on small molecules have been rarely observed and remain hardly explored^26^,^27^,^28^. Known examples of such materials show either a glass transition close to room temperature or the tendency to rapidly crystallize above the *T*_g_. Here we report a supramolecular glass that exhibits a high *T*_g_ and a low tendency to crystallize upon cooling from the melt. The design principles used here appear to be general and should permit easy access to other representatives of this interesting class of materials.

## Results

### Design and synthesis of a supramolecular glass

**Figure 1:**
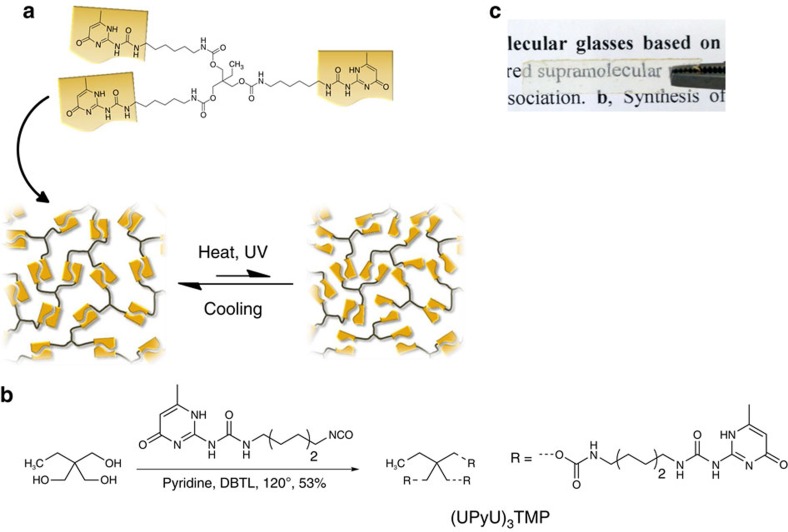
Supramolecular glasses based on (UPyU)_3_TMP. (**a**) Schematic representation of the formation of disordered supramolecular networks based on (UPyU)_3_TMP and their reversible, heat- or light-induced dissociation. (**b**) Synthesis of (UPyU)_3_TMP by the dibutyltindilaurate (DBTL) catalysed reaction of 1,1,1-tris(hydroxymethyl)propane with three equivalents of 2-(6-isocyanatohexylaminocarbonylamino)-6-methyl-4[1H]pyrimidinone in hot pyridine. (**c**) Picture of a self-supported (UPyU)_3_TMP film prepared by compression molding at 145 °C.

The optically responsive supramolecular material studied here is based on a trifunctional low-molecular-weight monomer (UPy functionalized 1,1,1-tris(hydroxymethyl)propane ((UPyU)_3_TMP)) that carries ureido-4-pyrimidinone (UPy) groups (Fig. 1a,b). The UPy motif, originally developed by Meijer and co-workers^29^, was chosen because it forms strong self-complementary hydrogen-bonded dimers, is easy to synthesize and its dynamic binding is well investigated^1^,^30^. Much of the previous work on UPy-based supramolecular polymers has focused on telechelic monomers with two terminal binding motifs that promote linear chain extension, and macromolecular systems in which UPy side chains serve as reversible cross-links^14^,^31^; these approaches afford supramolecular polymers whose properties are to a large extent governed by the nature of the telechelic or polymeric building blocks^32^. By contrast, the trifunctional (UPyU)_3_TMP introduced here was designed to form a supramolecular network whose properties are dictated by the cross-linked nature and the large content of the binding motif. We further surmised that the high concentration of the supramolecular motif, which tilts the dynamic equilibrium towards the bound state (Fig. 1a), and the cross-linked nature, which reduces the molecular mobility of the monomers, would hamper crystallization and permit kinetic trapping of a disordered amorphous glass upon cooling the material from a dissociated melted state^1^,^6^,^30^. Finally, we have shown recently that, if used in a sufficiently high concentration, the UPy motif can serve as an efficient light-heat converter^13^,^21^ and can be used to bestow polymers with optical responsiveness to permit features such as optical healing and (de)bonding on demand.

(UPyU)_3_TMP was prepared by reacting 1,1,1-tris(hydroxymethyl)propane with three equivalents of 2-(6-isocyanatohexylaminocarbonylamino)-6-methyl-4[1H]pyrimidinone using isocyanate chemistry (Fig. 1b). This simple reaction, which was carried out in hot pyridine to prevent network formation during the reaction and co-crystallization of solvent, as was observed when dimethylformamide was employed, afforded the new monomer in good yield. Precipitation of the product from the reaction mixture upon cooling afforded the product as analytically pure semi-crystalline powder, which upon melting turned into a clear, glassy material (Fig. 1c).

### Properties of (UPyU)_3_TMP

**Figure 2:**
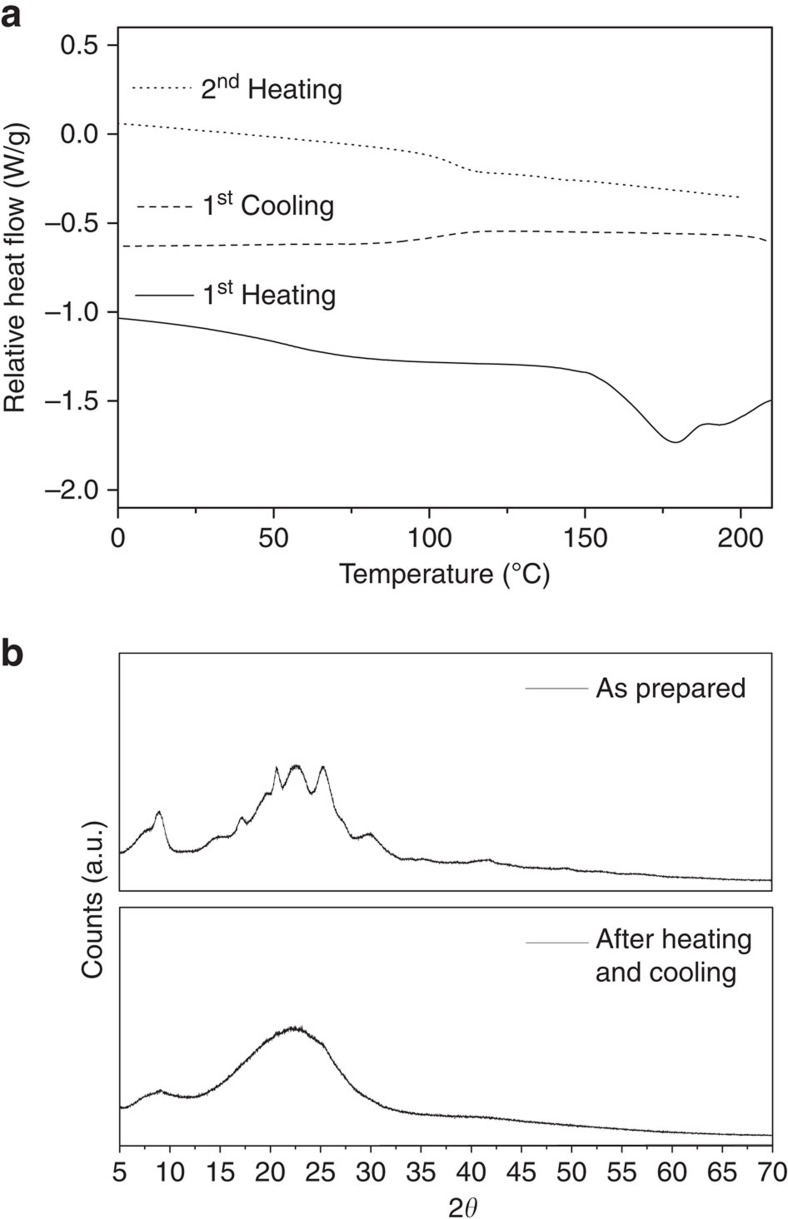
Thermal properties and morphology of (UPyU)_3_TMP. (**a**) Differential scanning calorimetry (DSC) traces (first heating (−), first cooling (- - -) and second heating (˙̇̇)) of the as-prepared material. The experiment was conducted with heating and cooling rates of 10 °C min^−1^ under N_2_ atmosphere; traces with other heating and cooling rates are provided as Supplementary Information. (**b**) Powder X-ray diffractograms for the as-prepared (top) material and a sample that had been heated to 200 °C and cooled to ambient.

**Figure 3:**
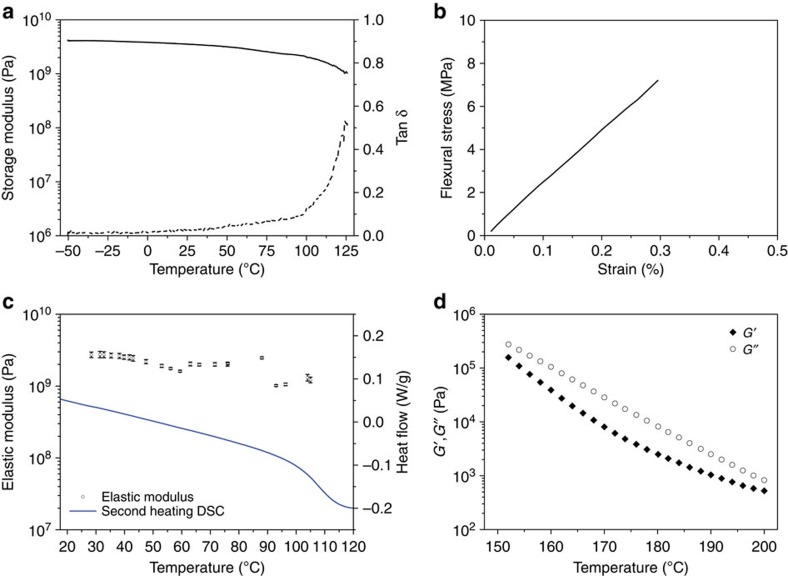
Mechanical and rheological properties of the (UPyU)_3_TMP supramolecular glass. (**a**) Representative dynamic mechanical analysis (DMA) trace of (UPyU)_3_TMP; shown are storage modulus (−) and tan *δ* (˙̇̇). (**b**) Representative flexural stress–strain curve of (UPyU)_3_TMP at 25 °C established by a three-point bending test. (**c**) Average surface elastic moduli (*n*=20) determined by AFM force spectroscopy as a function of temperature; also shown is the heat flow determined by DSC (second heating recorded at a rate of 10 °C min^−1^). (**d**) Storage (*G*′) and loss (*G*′′) moduli as function of temperature.

Thermogravimetric analysis (TGA) and differential scanning calorimetry (DSC) were used to determine the thermal properties of (UPyU)_3_TMP. The TGA of the as-prepared material (Supplementary Fig. 1) shows a 2% weight loss at 246 °C; above this temperature the decomposition rapidly accelerates. The DSC first heating trace of the as-prepared monomer shows a weak endothermic transition at 80 °C, which is associated with the glass transition, and an endothermic peak at 178 °C, corresponding to melting of the crystalline portion (Fig. 2a). The cooling scan reveals a glass transition around 100 °C and is void of any other transitions, even at a cooling rate as low as 1 °C min^−1^ (Supplementary Fig. 2), demonstrating that upon melting and cooling, (UPyU)_3_TMP forms a completely amorphous solid. This is confirmed by the second DSC heating trace, which also only shows a glass transition at 106 °C. Moreover, the DSC traces of (UPyU)_3_TMP glasses that were kept at ambient temperature for up to 7 months did not show any signs of crystallization upon storage (Supplementary Fig. 3). The interpretation of the DSC experiments was confirmed by powder X-ray diffraction experiments. The diffractogram of the as-prepared (UPyU)_3_TMP shows a superposition of an amorphous halo and well-defined reflections, whereas the diffractogram of a sample that had been heated to 200 °C and cooled to ambient temperature only displays diffuse diffraction (Fig. 2b). Taken together, these data indicate that the initially semicrystalline (UPyU)_3_TMP does not readily crystallize after being heated above its melting point; instead, the material forms a (kinetically trapped) amorphous glass, even when cooled very slowly.

(UPyU)_3_TMP can readily be melt-processed into solid supramolecular objects of various shapes by heating either the as-prepared crystalline monomer or material that had previously been converted into a glassy form to 200 °C (that is, above the melting temperature) to form a clear, slightly viscous liquid. Subsequent cooling to room temperature, optionally in a mold, affords a transparent hard material, for example, in the form of self-standing films (Fig. 1c) or coatings on substrates such as wood, glass or paper (Supplementary Fig. 4). Thin coatings on a paper substrate remained intact upon flexing the substrate (Supplementary Movie 1). Compression molding at 145 °C (5 tons, 5 s) afforded self-supporting films that were cut into rectangular shape (Fig. 1c) and sufficiently robust to allow for dynamic mechanical analysis (DMA) and three-point bending tests. The DMA trace of (UPyU)_3_TMP shows a glassy regime up to 125 °C with a room-temperature (25 °C) storage modulus of 3.65±0.51 GPa (Fig. 3a and Table 1), reflecting a very high stiffness. Three-point bending tests conducted at the same temperature confirmed the high stiffness (flexural modulus=3.04±0.26 GPa) and revealed a strain at break of 0.26±0.03% (Fig. 3b and Table 1) and a stress at break of 6.42±0.20 MPa. These data reflect that (UPyU)_3_TMP is quite brittle. We further probed the mechanical properties of the surface of a *ca.* 300-μm-thin (UPyU)_3_TMP film that had been melt-deposited on a glass substrate by depth-sensing indentation and atomic force microscopy (AFM) in force spectroscopy mode, in the latter case also as function of temperature. The room temperature Young's moduli—3.7±0.1 GPa measured by indentation (Supplementary Fig. 5) and 2.7±0.1 GPa measured by AFM (Fig. 3c)—are comparable to the value determined by DMA. Temperature-dependent AFM data reveal a significant modulus decrease upon heating, with an onset around 105 °C (Fig. 3c). A comparison with the DSC and DMA data makes evident that this stiffness decrease is associated with the transition from the glassy into a rubbery state. AFM force spectroscopy measurements also reveal a significant increase in adhesion above the *T*_g_ (Supplementary Fig. 6). The AFM data permit the conclusion that the UPy–UPy interactions are not simply ‘switched off' when the (UPyU)_3_TMP supramolecular glass is heated above *T*_g_; instead, a dynamic equilibrium between bound and dissociated states exists, which is shifted to the monomer side as the temperature is increased. Rheological studies were conducted to confirm this interpretation, which is consistent with the results of previous studies in which UPy dimerization was investigated as a function of temperature^14^. Frequency sweep experiments performed in the linear viscoelastic regime show a frequency dependence of the storage (*G*′) and loss (*G*′′) moduli between 150 and 200 °C (Supplementary Fig. 7), suggesting polymer-like viscoelastic properties. Further, a significant decrease of the *G*′ and *G*′′ was observed upon heating the material (Fig. 3d), suggesting a reduction of the virtual molecular weight on account of reducing the cross-link density by shifting the dynamic equilibrium towards the dissociated state.

**Figure 4:**
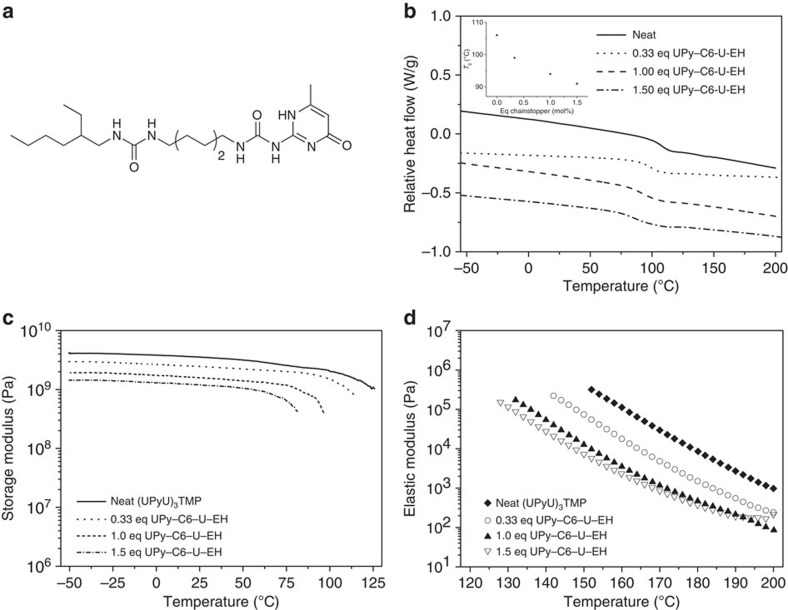
Influence of the addition of a chain stopper on the properties of (UPyU)_3_TMP. (**a**) Molecular structure of the chain stopper UPy-C6-U-EH. (**b**) Differential scanning calorimetry (DSC) traces. (**c**) Representative dynamic mechanical analysis (DMA) traces. (**d**) Elastic modulus as function of temperature. All samples were prepared by melt mixing (UPyU)_3_TMP with UPy-C6-U-EH at 200 °C and cooling to ambient.

The mechanical properties of (UPyU)_3_TMP are largely a result of the high cross-link density of the material in the solid state as well as above *T*_g_. We surmised that the introduction of a UPy-functionalized chain stopper would decrease the cross-link density and therefore influence the properties. To test this hypothesis, a mono-functional UPy-containing molecule was synthesized (2(6-(2-ethylhexyl)-8-hexylurea-aminocarbonylamino)-6-methyl-4[1H]pyrimidinone (UPy-C6-U-EH), Fig. 4a) and melt-mixed in various ratios with (UPyU)_3_TMP to form a series of amorphous supramolecular glasses (Supplementary Fig. 8). DSC experiments show that the *T*_g_ of these glasses decreases with increasing content of UPy-C6-U-EH (Fig. 4b and Supplementary Fig. 9).

DMA and three-point bending tests showed a decrease of the storage modulus, flexural modulus and stress at break upon addition of UPy-C6-U-EH (Fig. 4c and Table 1). The strain at break remained largely the same (Table 1), showing that a reduction of the cross-link density does not directly lead to a decrease in brittleness, arguably due to the glassy nature of the materials. Temperature-dependent AFM force spectroscopy data show the same trend for the stiffness of thin film surfaces (Supplementary Fig. 10). As expected, rheological studies show that increasing amounts of UPy-C6-U-EH cause a shift of the storage and loss modulus traces to lower temperatures (Fig. 4d). The DMA and rheology results are characteristic of a reduction of the cross-link density and confirm clearly that the addition of the UPy-C6-U-EH chain stopper has the desired significant effect.

### Stimuli-responsive behaviour of (UPyU)_3_TMP

**Figure 5:**
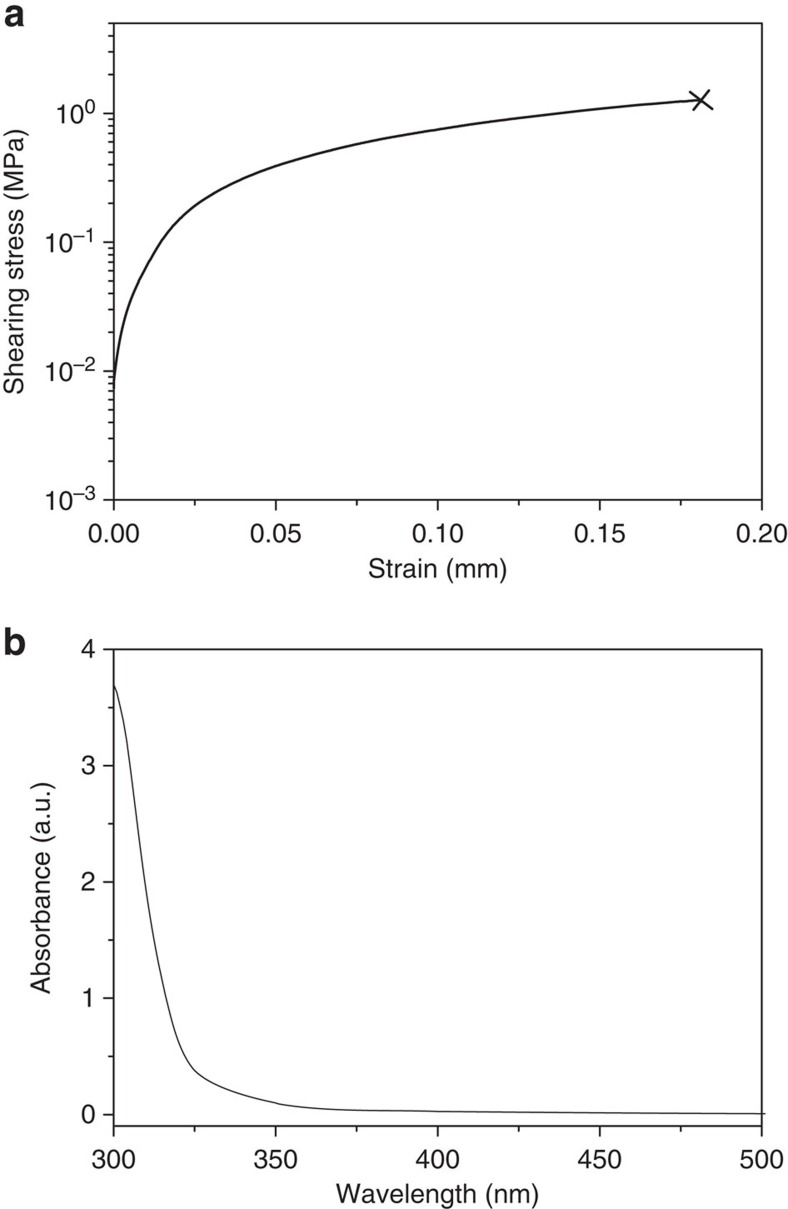
Adhesive and optical properties of (UPyU)_3_TMP. (**a**) Adhesion test of a lap joint formed by two glass slides that were bonded with (UPyU)_3_TMP (30 μm). (**b**) Ultraviolet–visible absorption spectrum of a solid film of (UPyU)_3_TMP (<30 μm) on a quartz substrate.

The targeted optically responsive nature of the new supramolecular glass relies on the conversion of (locally harnessed) optical energy into heat by non-radiative relaxation of the excited state. The rheological data presented show clearly that this causes the reversible dissociation of the network and formation of a melt. We first tested this by using (UPyU)_3_TMP as a reversible supramolecular adhesive. Single lap joints were prepared by joining two glass substrates, of which one was coated with a 30-μm-thin film of the glassy material, bonding them by heating to 200 °C for 10 s and cooling to ambient temperature (Supplementary Fig. 11). The lap joints displayed a shear stress of 1.2±0.2 MPa (Fig. 5a), which is comparable to that of other supramolecular adhesives^13^.

The optical absorption spectrum of amorphous (UPyU)_3_TMP shows an absorption band below 350 nm that can be attributed to the UPy motif (Fig. 5b)^21^. When the bonded lap joints were placed under load and exposed to ultraviolet light (*λ*=320–390 nm, 1,000 mW cm^−2^), the samples debonded within 30 s (Supplementary Movie 2). They could be re-bonded through exposure to light or heat, and the original adhesive properties were restored.

**Figure 6:**
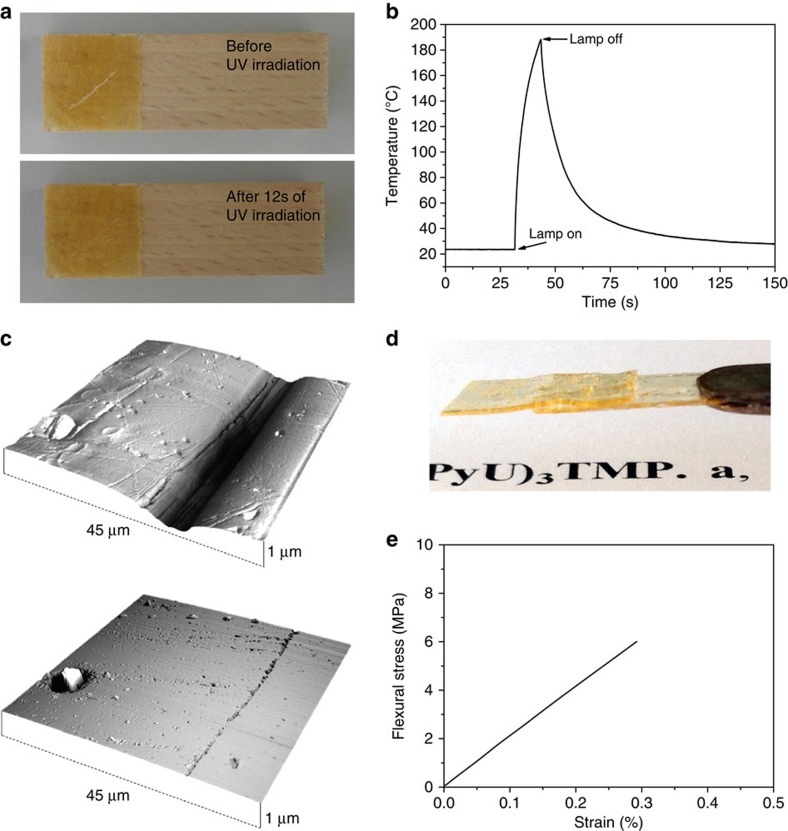
Optical healing of the (UPyU)_3_TMP supramolecular glass. (**a**) Pictures showing the optical healing of a damaged coating on wood. The 300-μm-thin coating was cut with a razor blade (top) and subsequently exposed to ultraviolet light for 12 s, which caused complete healing (bottom). (**b**) Surface temperature measured using an infrared camera upon irradiating the coating with ultraviolet light. (**c**) AFM images of damaged (top) and healed coating exposed to ultraviolet light for 12 s (bottom). (**d**) Picture of welded (UPyU)_3_TMP films; a film sample was cut in half, overlapped and irradiated with ultraviolet light (2 × 12 s). (**e**) Representative flexural stress–strain curve of a film mended as in **c** at 25 °C. An ultraviolet light source emitting at 320–390 nm and having a power density of 500 mW cm^−2^ (for experiments in **a**,**b**,**d**) or 650 mW cm^−2^ (for experiments in **c**,**e**) was used.

The high optical absorption imparted by the high UPy content (Fig. 5b), and the capability to dissociate into a low-viscosity melt should bestow the supramolecular (UPyU)_3_TMP glass with excellent optical healing capabilities. To test this, a piece of wood was coated with a 300-μm-thin layer of amorphous (UPyU)_3_TMP and the coating was intentionally damaged by cutting with a razor blade (Fig. 6a). The damaged area was subsequently exposed to ultraviolet irradiation (320–390 nm, 500 mW cm^−2^), which led to disappearance of the cut in as little as 12 s (Fig. 6a and Supplementary Movie 3). We monitored the temperature increase of the material with the help of an infrared thermometer; the data show a rapid and localized temperature increase to 188 °C (Fig. 6b). AFM images (Fig. 6c) show that a ca 10-μm-wide cut vanishes after 12 s of ultraviolet exposure, although a very shallow scar remained (Fig. 6c and Supplementary Fig. 12), whereas AFM force spectroscopy confirmed that the original and healed samples are identical in regard to their mechanical behaviour (Supplementary Fig. 13). The high transparency of (UPyU)_3_TMP also allowed optical welding of two films that were overlapped by irradiating such an assembly (320–390 nm, 650 mW cm^−2^, 2 × 12 s, Fig. 6d). The results of the three-point bending tests show no statistically significant difference from the original samples (Fig. 6e and Table 1) and samples were observed to always break outside of the mended area. We note that the healing and welding time depends on the light intensity and thermal conductivity of the substrate, which serves as a heat sink. Healing was also possible on glass (which has a higher thermal conductivity than wood), even when the power density was reduced to 250 mW cm^−2^ (Supplementary Fig. 14).

## Discussion

In summary, we have introduced a novel optically responsive glass-forming supramolecular material, which, in spite of the low-molecular-weight nature of the building block, displays typical polymeric behaviour, including high stiffness in the glassy state, viscoelastic behaviour in the melt and excellent coating and adhesive properties. Two specific characteristics appear to be particularly important in the context of the development of healable coatings. To our best knowledge, the supramolecular (UPyU)_3_TMP glass is not only stiffer than any other healable supramolecular polymer reported to date, but the material also heals exceedingly fast. This attractive combination of properties is a direct result of the design principle applied, that is, the use of a low-molecular-weight multifunctional building block to form a dynamic, disordered supramolecular network, which can readily be frozen into a glassy solid. The concept appears to be broadly applicable to other supramolecular glasses made of multifunctional monomers with binding motifs exhibiting sufficiently dynamic supramolecular interactions and light/heat conversion abilities. The most important limitation of the materials studied here vis-à-vis conventional thermosetting polymers are its limited toughness and the high brittleness, which may be improved by the adaptation of conventional toughening approaches^33^,^34^,^35^.

## Methods

### Reagents

All reagents were used as received. Unless indicated otherwise, all chemicals and solvents were obtained from Sigma-Aldrich and used as received. 3-Aminopropyl silica gel (0.6–1.3 mmol g^−1^) was acquired from TCI Japan. Anhydrous pyridine was purchased from Acros. Anhydrous dimethylformamide (DMF) was purified by passage through alumina columns. 2-(6-Isocyanatohexylaminocarbonylamino)-6-methyl-4[1H]pyrimidinone was synthesized as previously reported^36^.

### Characterization

^1^H (360 MHz) and ^13^C (90 MHz) NMR spectra were recorded on a Bruker Avance III spectrometer in dimethylsulphoxide (DMSO)-*d*6.^1^H NMR coupling constants are given in Hz. ^1^H NMR spectra were referenced against the signal of residual DMSO at 2.50 p.p.m. and ^13^C NMR spectra were referenced against the DMSO-*d*6 signal at 39.52 p.p.m. TGAs were conducted under N_2_ using a Mettler-Toledo STAR thermogravimetric analyser in the range of 25–500 °C with a heating rate of 10 °C min^−1^. DSC measurements were performed under N_2_ using a Mettler-Toledo STAR system operating at a heating/cooling rate of 10 °C min^−1^ in the range −70 to 150 °C, unless indicated otherwise. Data from the second heating cycle and the reverse heat flow curve are reported unless indicated otherwise (*T*_g_=glass transition temperature). DMAs were performed on a TA Instruments DMA Q800 with a heating rate of 5 °C min^−1^ and a frequency of 1 Hz in the range of −50 to 140 °C using a three-point bending setup. Three-point bending tests were conducted on the same instrument at 25 °C, with a displacement rate of 100 μm min^−1^. All mechanical tests were conducted on rectangular samples (typical dimensions: 25 mm × 5 mm × 230 μm). Flexural moduli were determined from the entire curves, as all samples displayed a linear relation between stress and strain until failure. Load-sensing indentation measurements were performed using a CSM Ultra Nanoindenter equipped with a Berkovich tip (diamond). All experiments were performed using a loading and unloading rate of 100 μN min^−1^, and 15 min of constant load (100 μN) before unloading to allow for creep deformation. AFM images and force spectroscopy measurements were performed on a JPK Nano Wizard II. AFM images were recorded with NanoWorld NCHR high-resonance frequency tips. Force spectroscopy tests were performed with a Bruker DNISP cantilever with a cube corner diamond tip (nominal sensitivity=249 N m^−1^). Temperature-dependent AFM force spectroscopy tests were performed on coatings with a thickness of 300 μm on a thin round microscopy glass slide and placed on a JPK HTHS high-temperature heating stage, and the sample surface temperature was continuously monitored using a hand-held infrared camera. All recorded unloading curves were fitted (upper 50% of the unloading curve) and analysed according to the Oliver and Pharr model to yield the elastic modulus, assuming a Poisson ratio of 0.3 and a perfect cube corner tip^37^. Rheological studies were performed on a TA instruments ARG2 Rheometer operating with a Peltier heating stage and parallel plate geometry. Ultraviolet–visible absorption spectra were measured on a Jasco V-670 spectrophotometer. Electrospray ionization mass spectrometry (ESI-MS) was measured by the Laboratory for Mass Spectroscopy of the University of Fribourg. Elemental analysis was performed by the Service d'analyses chimiques of the Ecole d'ingénieurs et d'architectes of Fribourg. FT-IR spectra were measured on a Perkin-Elmer Spectrum 65 spectrometer using dried (crystalline) powder in attenuated total reflection (ATR) mode between 800 and 4,000 cm^−1^ with five accumulated scans per sample. Ultraviolet irradiation of the samples to achieve healing was performed with a Hoenle Bluepoint 4 Ecocure lamp connected to an optical fibre. An optical filter was used to irradiate limit the output to the wavelength range of 320–390 nm. The temperature increase during ultraviolet irradiation was monitored using an Optris PI connect infrared camera. Adhesion tests were performed by adhering two regular glass slides (overlapping area=10 × 25.7 mm^2^) with a thin layer of (UPyU)_3_TMP). The adhesive properties were determined with a Zwick/Roell Z010 tensile tester at room temperature with a strain rate of 0.1 mm min^−1^.

### Synthetic methods

*Synthesis of (UPyU)_3_TMP*. For the synthesis of (UPyU)_3_TMP, a round bottom flask equipped with a reflux cooler was charged with 1,1,1-tris(hydroxymethyl)propane (635 mg, 4.7 mmol), 2-(6-isocyanatohexylaminocarbonylamino)-6-methyl-4[1H]pyrimidinone (5.0 g, 17 mmol), dibutyltin dilaurate (300 μl, ∼3 mol%) and dry pyridine (300 ml) under an N_2_ atmosphere. The reaction mixture was heated to reflux temperature (120 °C) and was stirred for 36 h under reflux. Aminopropyl functionalized silica (5 g) was added to eliminate any excess of the UPy isocyanate. The reaction mixture was stirred for an additional 30 min at reflux temperature to prevent precipitation of the product. The reaction mixture was cooled to 100 °C and solids were removed from the reaction mixture by vacuum filtration. The filtrate was cooled to room temperature and a white precipitate was observed. Acetone (200 ml) was added to the filtrate, and the solids were collected by filtration. The precipitate was washed with acetone (3 × 100 ml) and dried *in vacuo* at 70 °C for 12 h to yield the analytically pure product as white crystalline powder (3.7 g, 3.6 mmol, 53%). We note that the product has poor solubility in common solvents, therefore all NMR samples were prepared by heating to 100 °C in DMSO-*d*6 for 20 min to achieve complete dissolution (*c*=2 mg ml^−1^). Cooling to room temperature resulted in solution that were stable for up to 2 h. ^1^H NMR (DMSO-*d*6, 360 MHz) *δ*=11.53 (s, 3H), 9.67 (s, 3H), 7.39 (s, 3H), 7.09 (s, 3H), 5.76 (s, 3H), 3.85 (ds, 6H), 3.12 (t, 6H), 2.93 (s, 6H), 2.10 (s, 9H), 1.43–1.27 (bm, 26H), 0.8 (s, 3H). ^13^C NMR (CDCl_3_, 91 MHz) *δ*=173.67, 158.98, 156.99, 155.13, 148.73, 147.08, 107.05, 103.68, 41.11, 40.10, 36.59, 30.56, 29.85, 26.75, 19.35. ESI-MS: *m/z*: calculated: 1,014.1; found: 1,014.2. Analysis calculated for C_45_H_71_N_15_O_12_: C=53.29, H=7.06, N=20.72. Found: C=53.30, H=7.46, N=20.80.

*Synthesis of UPy-C6-U-EH*. For the synthesis of UPy-C6-U-EH, a round bottom flask was charged with 2-ethylhexyl-1-amine (559 μl, 3.4 mmol), 2-(6-isocyanatohexylaminocarbonylamino)-6-methyl-4[1H]pyrimidinone (1.0 g, 3.4 mmol) and dry DMF (25 ml) under an N_2_ atmosphere. The reaction mixture was heated to 90 °C for 12 h, and subsequently cooled to room temperature. The mixture was diluted with diethyl ether (100 ml), and a white precipitate was formed. The precipitate was filtered, washed with diethyl ether (2 × 50 ml) and dried *in vacuo* at 50 °C for 12 h to yield the pure product as white crystalline powder (1.3 g, 3.1 mmol, 90%). ^1^H NMR (DMSO-*d*6, 360 MHz) *δ*=11.56 (s, 1H), 9.66 (s, 1H), 7.34 (s, 1H), 5.77 (s, 1H), 5.71 (q, 2H), 3.13 (q, 2H), 2.95 (dq, 4H), 2.10 (s, 3H), 1.44–1.25 (bm, 17H), 0.81 (dt, 6H). ^13^C NMR (101 MHz, DMSO) *δ*=158.67, 42.32, 30.88, 30.48, 29.58, 28.89, 26.48, 26.45, 24.10, 23.01, 14.44, 11.27. ESI-MS: *m/z*: calculated: 422.3; found: 423.3 [M+1]^+^.

### General methods

*Preparation of thin coatings and self-standing films*. As-prepared (UPyU)_3_TMP was heated on a microscopy glass for 5 min at 200 °C to yield a clear viscous material. The material was transferred with a spatula onto a preheated glass slide and doctor-bladed to form a homogenous film. To form a coating on wood, the viscous liquid material was placed on a hot microscopy glass slide and doctor-bladed onto the wood surface to achieve a homogenous coating. This process was repeated to yield a coating of around 300 μm thickness. Self-standing films were prepared by first heating the (UPyU)_3_TMP powder to 200 °C in a vial and cooling the resulting viscous liquid to room temperature. The resulting amorphous material was compression molded in a Carver press between two Kapton sheets that were separated by Teflon spacers (145 °C, 5 s at a pressure of 5 tons after the material had liquefied). Large crack formation was observed when samples were cooled to room temperature between the Kapton sheets. To produce films without macroscopic cracks, the assembly was removed from the hot press, and as soon as the (UPyU)_3_TMP had reached the temperature at which it had solidified into a clear, slightly yellow film, the Kapton liners were removed. The samples were subsequently cooled to room temperature. To prepare rectangular samples for mechanical testing, (UPyU)_3_TMP films were placed on a hotstage that was heated to 145 °C and lined with a Kapton film, before they were cut using a sharp razor blade. To minimize the influence of environmental conditions, mechanical tests were conducted within a day after producing the samples.

*Indentation measurements*. For indentation measurements, a glassy (UPyU)_3_TMP coating with a thickness of around 300 μm on a thin round glass substrate was prepared by melt deposition at 200 °C onto a thin glass substrate. The coating thickness was chosen with respect to Buckle's one-tenth law to avoid any influence of the substrates on the measurement^38^, and maximum indentation depths did not exceed 3 μm (<1%). Furthermore, as a control experiment to improve the reliability of the fitted data, AFM images of indents (for indentation forces of 150 and 300 μN) were acquired. Perfect cube corner indents with only limited pile-up (Supplementary Fig. 15) are seen, especially for indents with a force of 150 μN, which was used for all AFM force spectroscopy experiments. We note that fitted data from nano indentation measurements yield a comparative impression of the order magnitude of stiffness, and do not give absolute values for the stiffness. Depth-sensing indentation measurements were performed by indentation at room temperature with a maximum force of 100 μN at a loading and unloading rate of 100 μN min^−1^. Before each measurement, a height calibration of the local sample surface was performed. Unloading curves were used to determine the elastic modulus according to the Oliver and Pharr model using CSM nanoindentation software^37^. AFM force spectroscopy measurements were carried out by using a cube corner diamond tip cantilever. An indentation force of 150 μN was chosen to respect the indentation depth limitation dictated by Buckle's Law regarding effects of the substrate. Data were then analysed using the Oliver and Pharr model with a Poisson ratio of 0.3 and the slope of the initial part (that is, upper 50%) of the unloading indentation curve.

*Rheology*. All rheological studies were carried out using parallel plate geometry and controlled normal force (between −0.5 and 0.5 N). Samples were measured by heating (UPyU)_3_TMP or mixtures of (UPyU)_3_TMP and (UPy-C6-U-EH) to 200 °C for 2 min before the rotating parallel plate was lowered until the material was observed to flowed out on all sides of the upper plate; subsequently, excess material was removed to prevent edge effects on the data. Frequency sweep experiments were conducted at fixed temperature intervals between 200 and 90 °C at a strain of 0.2% and a frequency of 20 rad s^−1^. The data were analysed with TA instruments software, and further processed with graphing software. Data were rejected when the strain on the sample was <75% of the strain applied.

*Chain stopper experiment*. (UPyU)_3_TMP and varying amounts of UPy-C6-U-EH (0.33, 1.0 and 1.5 molecular equivalents) were mixed as powders and then heated to 200 °C for 15 min to yield a clear homogenous melt. Coatings and films for AFM force spectroscopy measurements, DMA and three-point bending tests were prepared as described above for the neat (UPyU)_3_TMP.

*Adhesive properties and optical debonding*. The adhesive properties of (UPyU)_3_TMP on robust glass substrates were determined with a tensile tester at room temperature with a strain rate of 0.1 mm min^−1^. To do that, two glass slides were joined by a 30-μm-thick layer of (UPyU)_3_TMP at 200 °C and subsequent cooling to room temperature. For optical debonding experiments, glass slides (thickness=140 μm) were joined together with (UPyU)_3_TMP in the same manner, and one side of the resulting lap joint was mounted into a holder, whereas a weight of 500 g was attached to the glass slide. Debonding was achieved by irradiation with ultraviolet light (320–390 nm 1,000 mW cm^−2^). This experiment was conducted with thin glass slides to limit heat dissipation.

*Optical healing*. Optical healing of scratches was performed on coatings of (UPyU)_3_TMP (thickness=300 μm) that were damaged with a razor blade. Healing was achieved by irradiation with ultraviolet light (320–390 nm, 500 mW cm^−2^, 12 s) and the temperature was monitored by an infrared camera. After 12 s, a small scar is still visible, and can be completely removed by another 12 s irradiation with the same intensity. For optical mending rectangular pieces of (UPyU)_3_TMP films were cut into two with a razor blade. The samples were overlapped and without external pressure irradiated with ultraviolet light (320–390 nm, 650 mW cm^−2^, 2 × 12 s with 5 min between the exposures to allow for cooling to room temperature).

## Additional information

**How to cite this article:** Balkenende, D. W. R. *et al*. Optically responsive supramolecular polymer glasses. *Nat. Commun.* 7:10995 doi: 10.1038/ncomms10995 (2016).

## Supplementary Material

Supplementary Information Supplementary Figures 1-17.

Supplementary Movie 1 A flexible coating of (UPyU)3TMP on paper. A coating (thickness < 50 μm) of (UPyU)3TMP was applied on paper from the melt at 200 °C. Flexing the coating on paper does not result in the formation of cracks.

Supplementary Movie 2 Optical debonding on demand of (UPyU)3TMP as adhesive. Lap joint were formed by overlapping two thin glass slides and bonding them with a thin layer of (UPyU)3TMP. The movie shows debonding of a lap joint that was placed under load and exposed to ultraviolet light (Λ = 320-390 nm, 1000 mW cm^−2^).

Supplementary Movie 3 Optical healing of a coating of (UPyU)3TMP. The movie shows a 300 μm thin coating that was cut with a razor blade and subsequently exposed to UV light for 12 s.

## Figures and Tables

**Table 1:** Mechanical properties of (UPyU)_3_TMP and (UPyU)_3_TMP(UPy-C6-U-EH)_*x*_.

Sample	Storage modulus (GPa)*	Flexural modulus (GPa)†	Flexural stress at break (MPa)†	Flexural strain at break (%)†
(UPyU)_3_TMP	3.65±0.51	3.04±0.26	6.42±0.20	0.26±0.03
(UPyU)_3_TMP(UPy-C6-U-EH)_0.33_	2.87±0.36	2.08±0.28	6.41±0.96	0.30±0.03
(UPyU)_3_TMP(UPy-C6-U-EH)_1.0_	2.21±0.77	1.68±0.14	5.58±0.95	0.33±0.09
(UPyU)_3_TMP(UPy-C6-U-EH)_1.5_	1.47±0.29	1.29±0.12	4.44±0.83	0.34±0.06
(UPyU)_3_TMP healed‡	NA	2.80±0.43	4.81±2.74	0.27±0.15

Abbreviation: NA, not applicable; UPy, ureido-4-pyrimidinone; (UPyU)_3_TMP, UPy functionalized 1,1,1-tris(hydroxymethyl)propane; UPy-C6-U-EH, 2(6-(2-ethylhexyl)-8-hexylurea-aminocarbonylamino)-6-methyl-4[1H]pyrimidinone.

^*^Measured by DMA at 25 °C with *n*=3 individual measurements.

^†^Measured by three-point bending stress–strain experiments at 25 °C, *n*=5 (except for healed samples, where *n*=10).

^‡^Samples were cut in half, partly overlapped and irradiated with ultraviolet light (320–390 nm, 650 mW cm^-2^, 2 × 12 s).
