# Supplementary material for: Optically responsive supramolecular polymer glasses
Source: Nat Commun. 2016 Mar 17;7:10995. doi: 10.1038/ncomms10995 (PMC4800438; doi:10.1038/ncomms10995)
Supplement: Supplementary Information — Supplementary Figures 1-17. [file ncomms10995-s1.pdf]

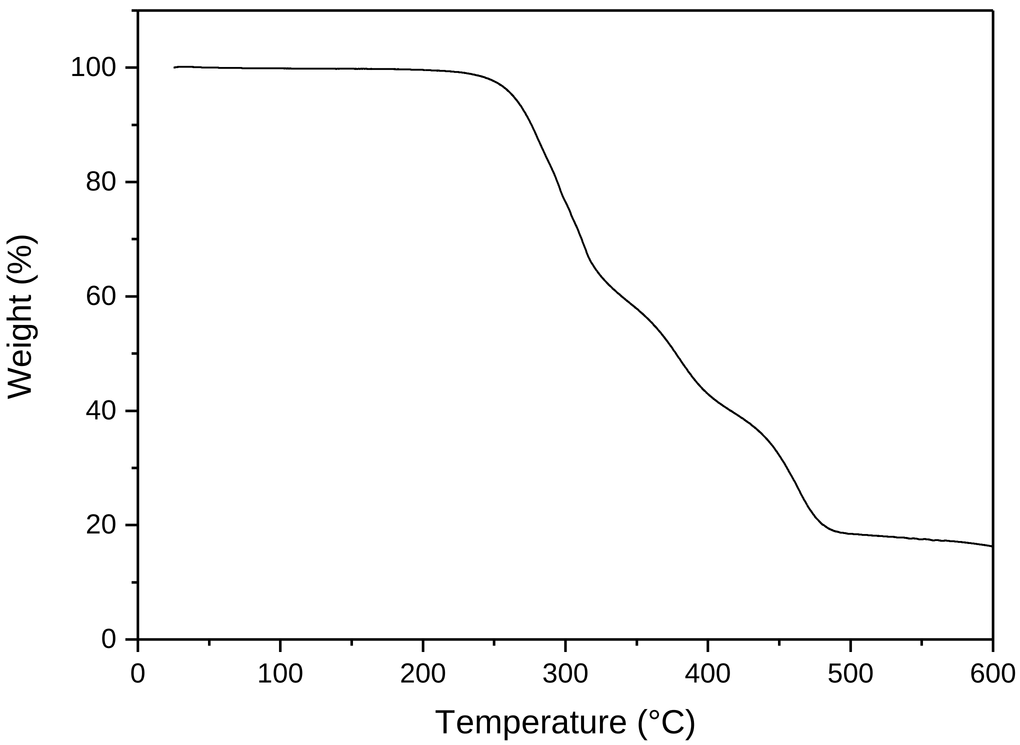

Supplementary Figure 1. Thermal analysis of $(UPyU)_3$TMP. Thermogravimetric analysis (TGA) curve of $(UPyU)_3$TMP from 25 to 600 °C. The experiment was performed at a heating rate of 10° C·min^{-1} under N_2 atmosphere.

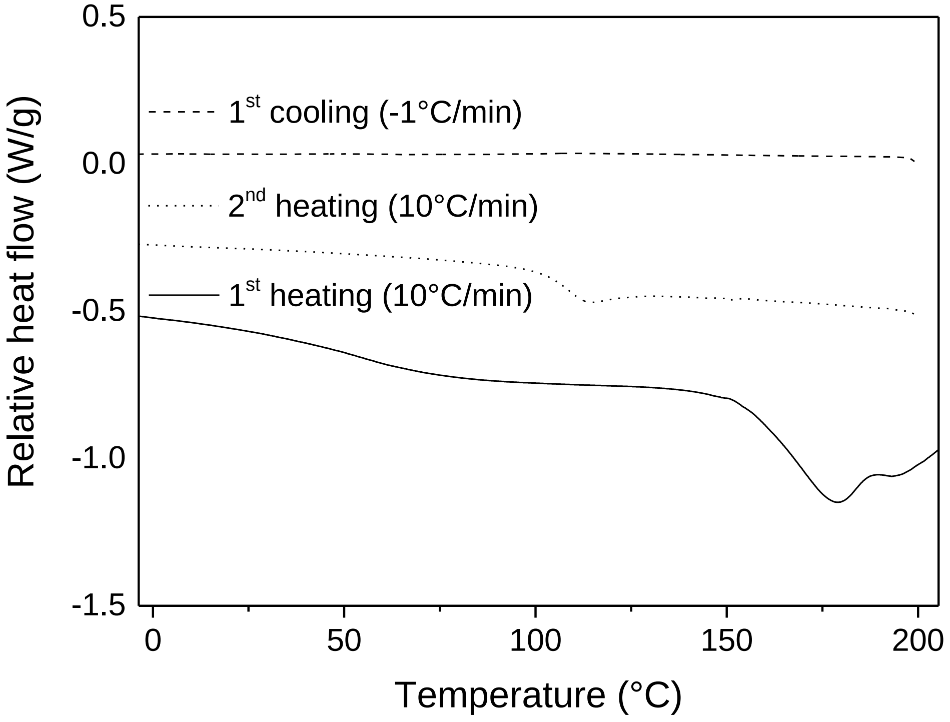

Supplementary Figure 2. Thermal analysis of (UPyU)$_3$TMP. Differential scanning calorimetry (DSC) curves of (UPyU)$_3$TMP; first heating (—), first cooling (- - -) and second heating (···). The experiment was performed with heating and cooling rates of 10° C·min^{-1} for the first and second heating, and the first cooling at 1° C·min^{-1}. No crystallization was observed in the first cooling or second heating, indicating that the material forms an amorphous material even at low cooling rates.

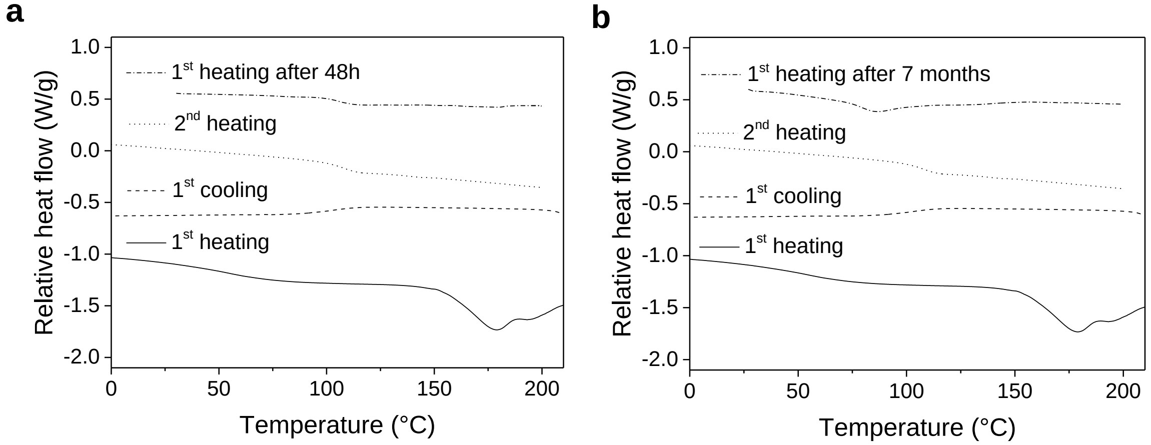

Supplementary Figure 3. Ageing of amorphous (UPyU)$_3$TMP. Differential scanning calorimetry (DSC) traces showing first heating (—), first cooling (- - -), second heating (···) of as prepared (UPyU)$_3$TMP together with the traces of samples that were stored for **a,** 48 hours (-.-) or **b,** 7 months (-.-). For the ageing experiment in **a** (UPyU)$_3$TMP was heated to 200 ºC, cooled to ambient and then stored in a dry atmosphere for 48 hours prior to the DSC measurement. In the case of **b** (UPyU)$_3$TMP was heated to 200 ºC, cooled to ambient and then stored under ambient conditions for 7 months. To eliminate any water that the sample might have absorbed during storage, the sample was dried at room temperature under high vacuum for 48 hours prior to the DSC measurement. DSC experiments were performed with heating and cooling rates of 10º C·min^{-1} under a N_2 atm.

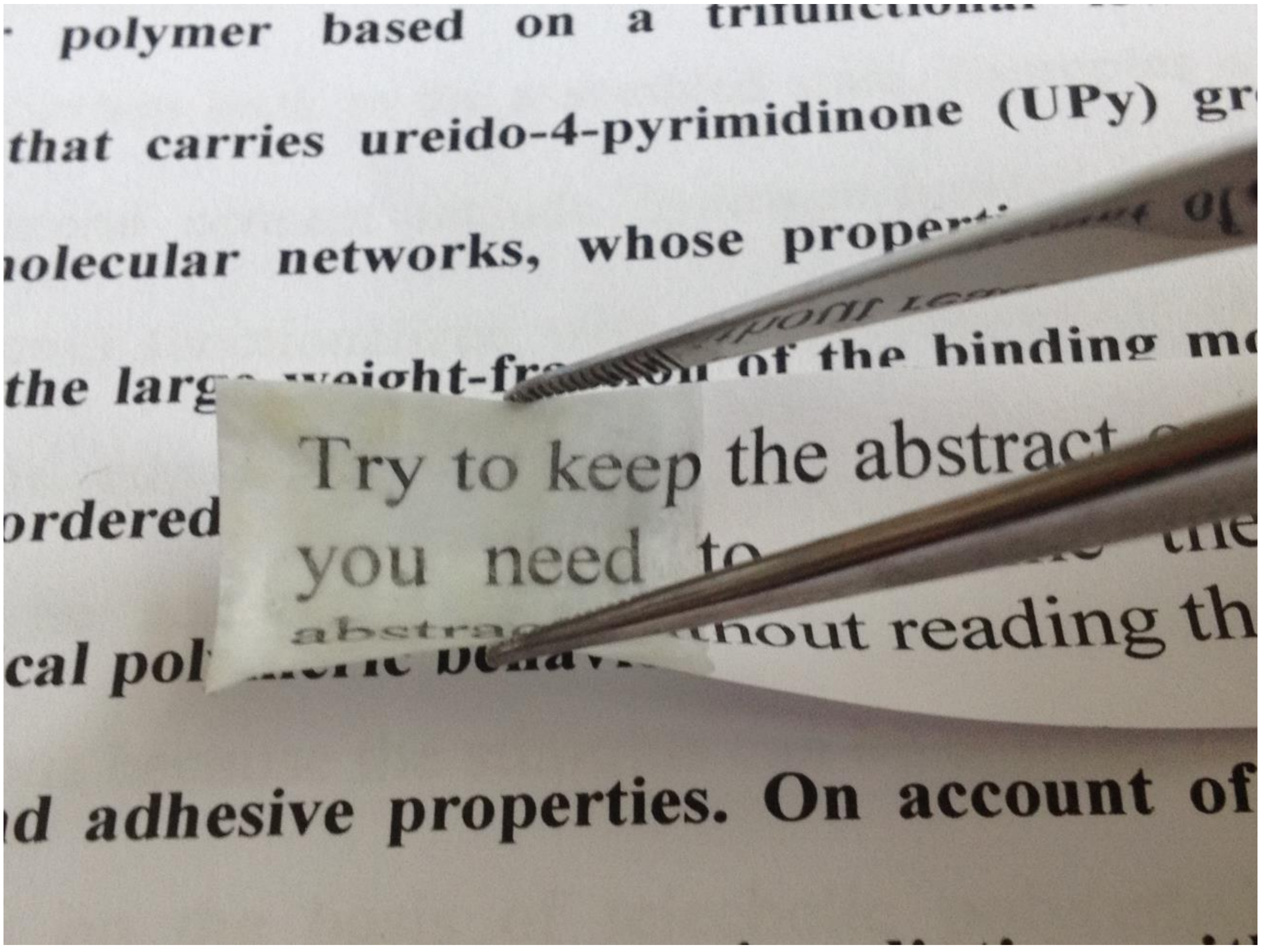

Supplementary Figure 4. Image of a flexible coating of $(UPyU)_3TMP$ on paper. A coating (thickness < 50 µm) of $(UPyU)_3TMP$ was applied on paper from the melt at 200 ºC. Flexing the coating on paper does not result in the formation of cracks.

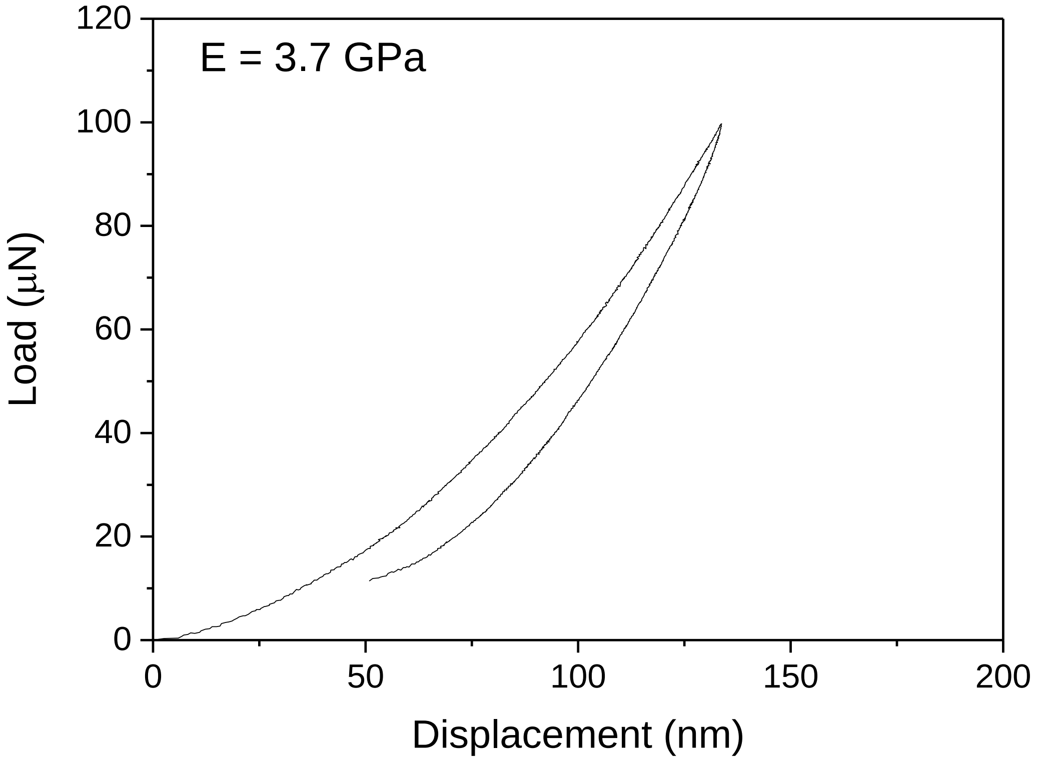

Supplementary Figure 5. Indentation measurement of $(UPyU)_3TMP$ by depth sensing indentation. Load displacement curve acquired by depth sensing indentation. A fit of the unloading curve resulted a surface elastic modulus of 3.7 GPa.

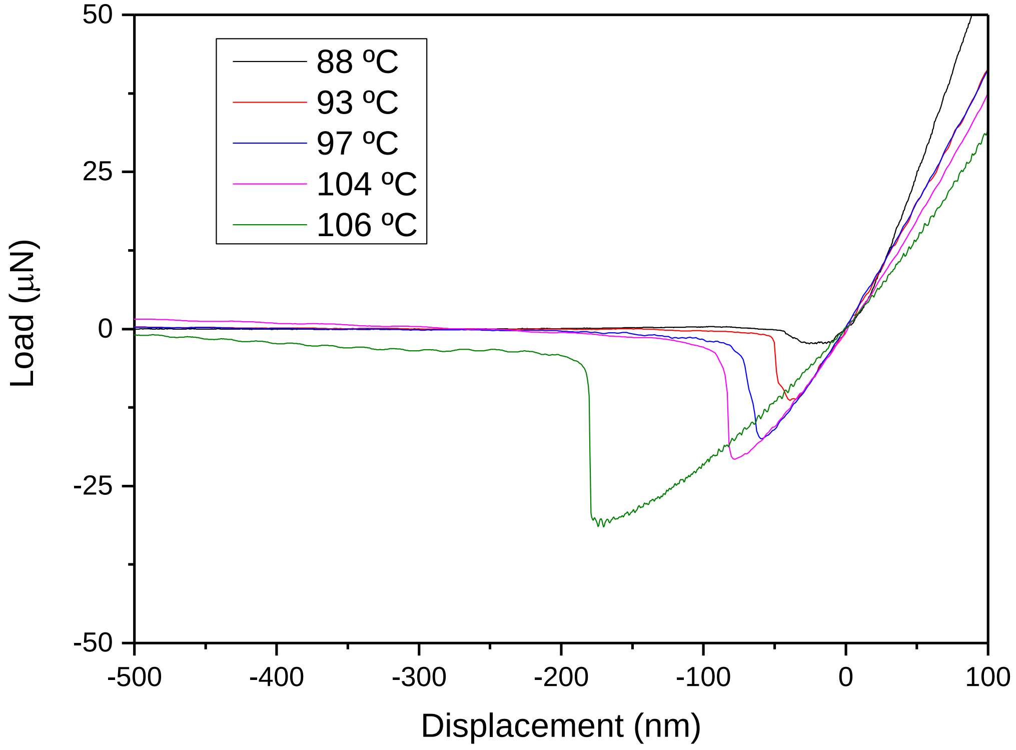

Supplementary Figure 6. Indentation measurements of $(UPyU)_3TMP$ by force spectroscopy. Magnification of the unloading part of the force displacement curves of $(UPyU)_3TMP$ at different temperatures. A negative load in the negative displacement region indicates adhesion of $(UPyU)_3TMP$ during unloading. An increase of adhesion was observed upon heating towards T_g (107 ºC). Above 106 ºC, the sample became too soft to measure with the DNSIP cantilever.

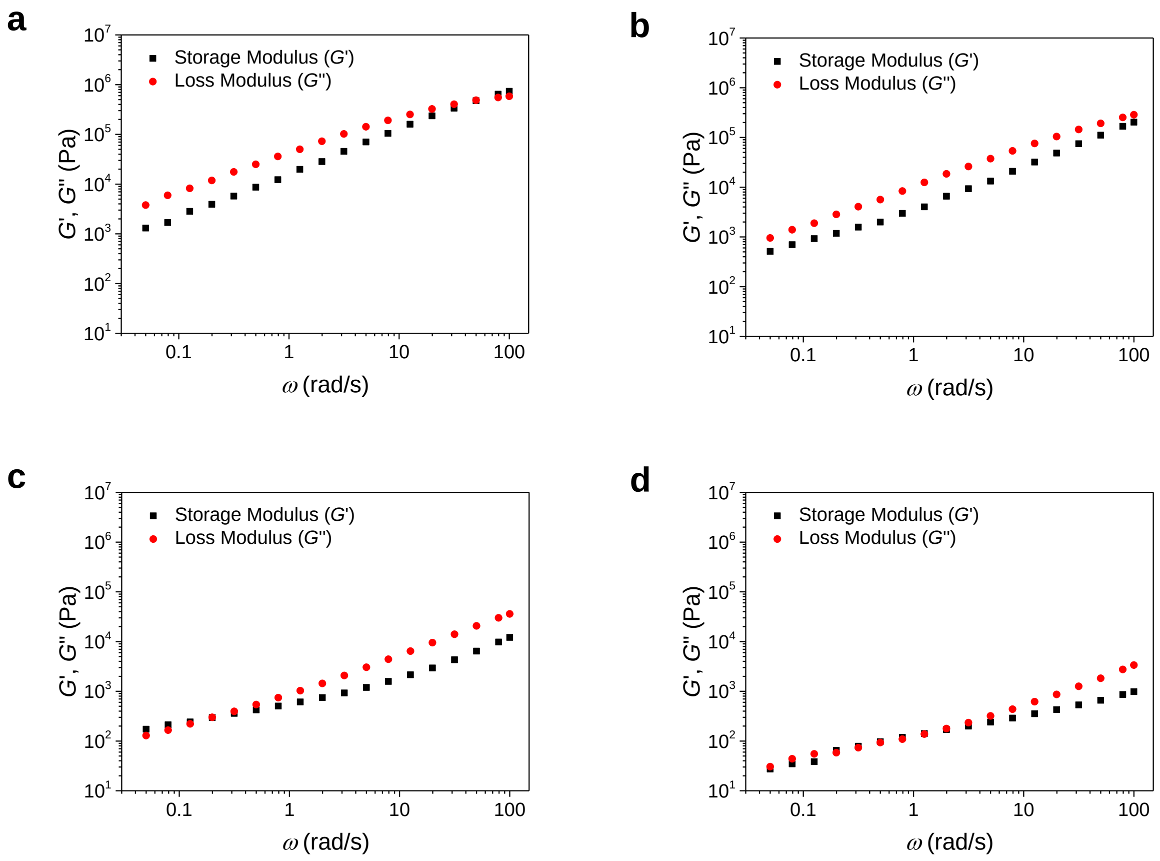

Supplementary Figure 7. Mechanical analysis of (UPyU)$_3$TMP by rheology. Frequency sweep experiments of (UPyU)$_3$TMP yielding the storage (G') and loss modulus (G") as function of the frequency (ω, rad/s) were performed at **a**, 150 ºC; **b**, 160 ºC; **c**, 180 ºC; and **d**, 200 ºC. An increase of G' and G" as function of the rotational frequency is associated with a viscoelastic material.

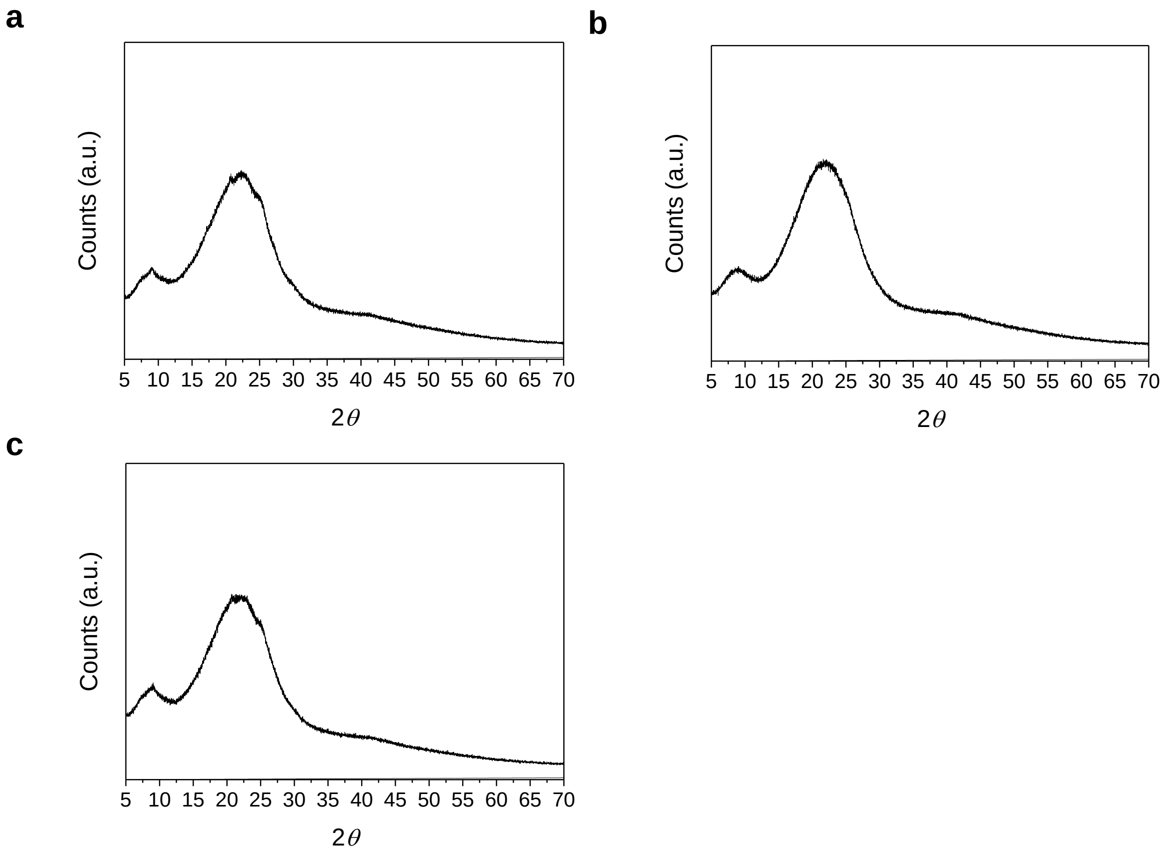

Supplementary Figure 8. Powder x-ray diffraction of mixtures of $(UPyU)_3$TMP and UPy-C6-U-EH. Shown are the diffractograms of **a**, $(UPyU)_3$TMP$(UPy\text{-}C6\text{-}U\text{-}EH)_{0.3}$; **b**, $(UPyU)_3$TMP$(UPy\text{-}C6\text{-}U\text{-}EH)_{1.0}$; **c**, $(UPyU)_3$TMP$(UPy\text{-}C6\text{-}U\text{-}EH)_{1.5}$. Diffuse diffraction confirms that all three mixtures are amorphous.

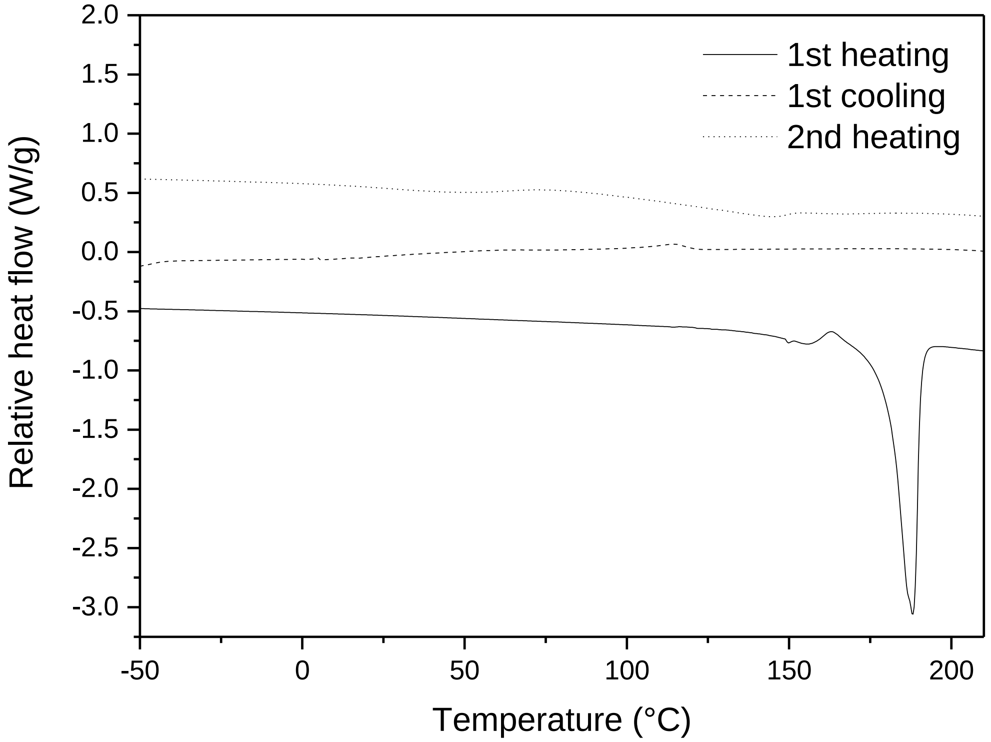

Supplementary Figure 9. Thermal analysis of UPy-C6-U-EH. Modulated differential scanning calorimetry (DSC) curves of UPy-C6-U-EH; first heating (—), first cooling (- - -) and second heating (···). The experiment was performed with heating and cooling rates of 10º C·min^{-1} under a N_2 atmosphere.

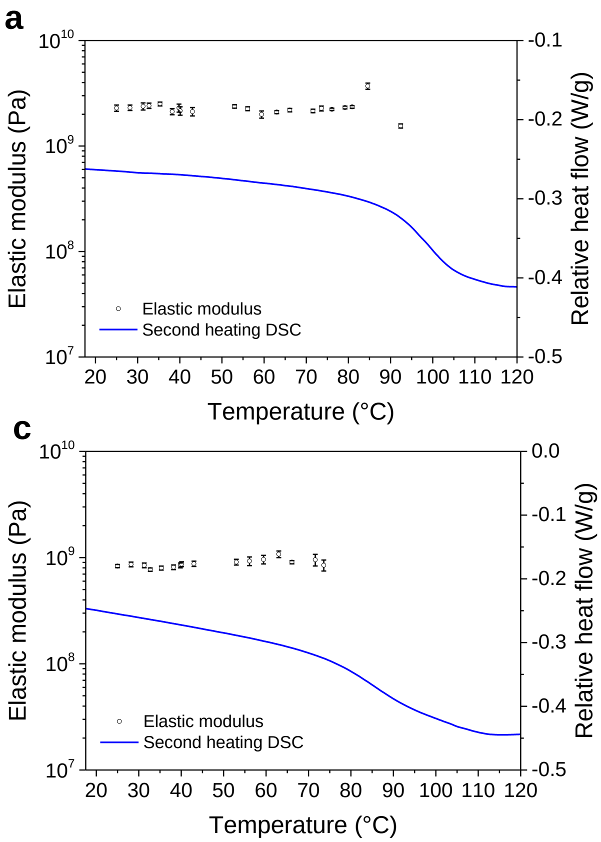

Supplementary Figure 10. Indentation measurements of mixtures of $(UPyU)_3TMP$ and (UPy-C6-U-EH) by force spectroscopy. Average surface elastic moduli (n = 20) of **a**, $(UPyU)_3TMP$ and 30 mol% of (UPy-C6-U-EH); **b**, $(UPyU)_3TMP$ and 100 mol% of (UPy-C6-U-EH); **c**, $(UPyU)_3TMP$ and 150 mol% of (UPy-C6-U-EH) determined by AFM force spectroscopy as a function of temperature; also shown is the heat flow determined by DSC (second heating recorded at a rate of 10 °C·min^{-1}).

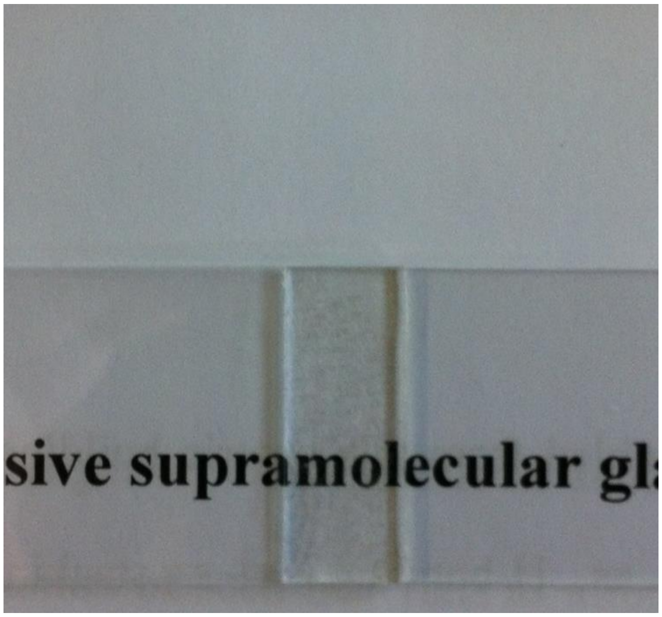

Supplementary Figure 11. Use of $(UPyU)_3TMP$ as a reversible adhesive. The image shows a lap joint formed by overlapping two glass slides (overlap area: 1.0 cm x 2.5 cm) and bonding them with a thin layer of $(UPyU)_3TMP$. The overlapped microscopy slides were used in stress strain experiments to determine the adhesive strength of $(UPyU)_3TMP$.

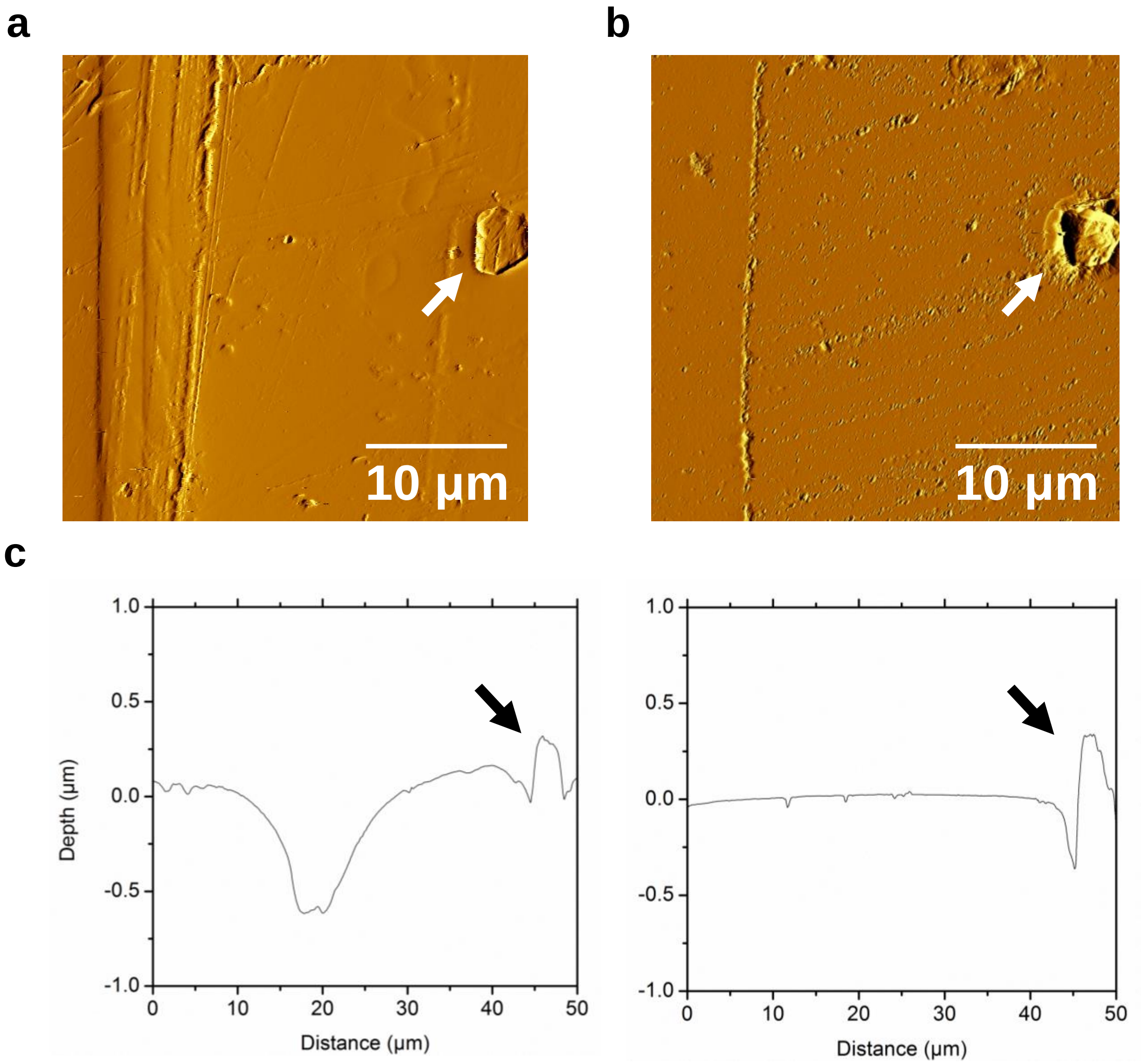

Supplementary Figure 12. Optical healing of the $(UPyU)_3TMP$ supramolecular glass recorded by AFM. Vertical deflection images of **a**, deliberately damaged sample and **b**, the same sample area after 12 s of UV irradiation (320 – 390 nm 500 mW·cm^{-2}). **c**, Corresponding height profiles before and after optical healing. The arrows indicate a bubble that served as point of reference.

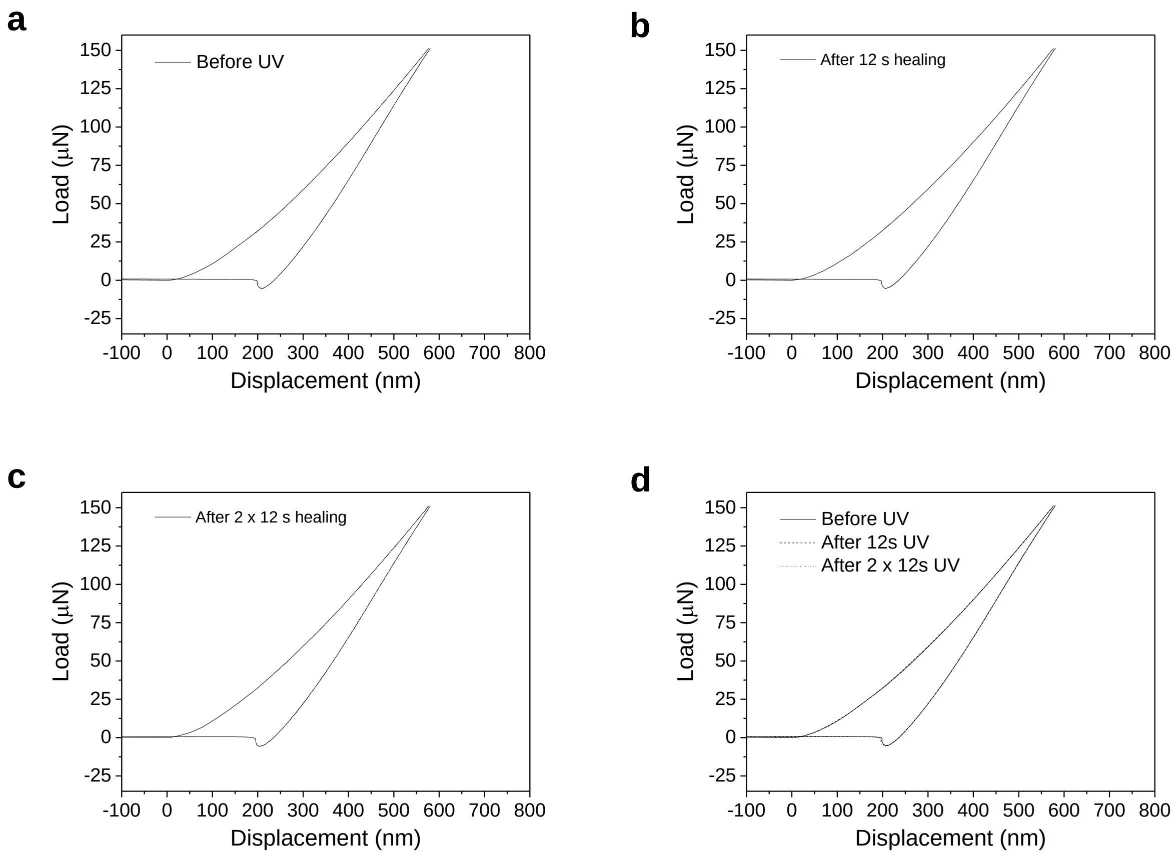

Supplementary Figure 13. Optical healing of the (UPyU)$_3$TMP supramolecular glass followed by AFM force spectroscopy. Representative loading and unloading curves of **a**, before irradiation with UV light; **b**, after 12 sec of UV irradiation (320 – 390 nm 500 mW·cm^{-2}); **c**, after 2 times 12 sec of UV irradiation; and **d**, combined curves. The exact overlap of all loading and unloading curves indicates that material properties are not compromised by damage or UV irradiation. Force curves after healing were measured 10 min after irradiation with UV light to allow the samples to cool down to room temperature.

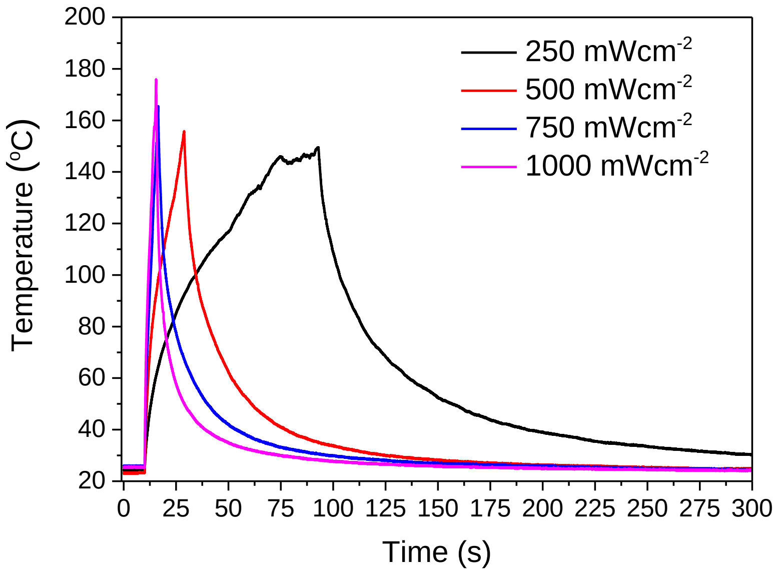

Supplementary Figure 14. Optical healing of (UPyU)$_3$TMP. Surface temperature measured using an IR camera upon irradiating a coating of (UPyU)$_3$TMP on a glass substrate (thickness 140 µm) with UV irradiation (320 – 390 nm) as function of intensity. To probe healing, the sample were cut with a razor blade and subsequently exposed to UV light and the time until the cut had completely disappeared was monitored and found to be ca. 80, 20, 7, and 5.5 s for intensities of 250, 500, 750 and 1000 mW/cm^2.

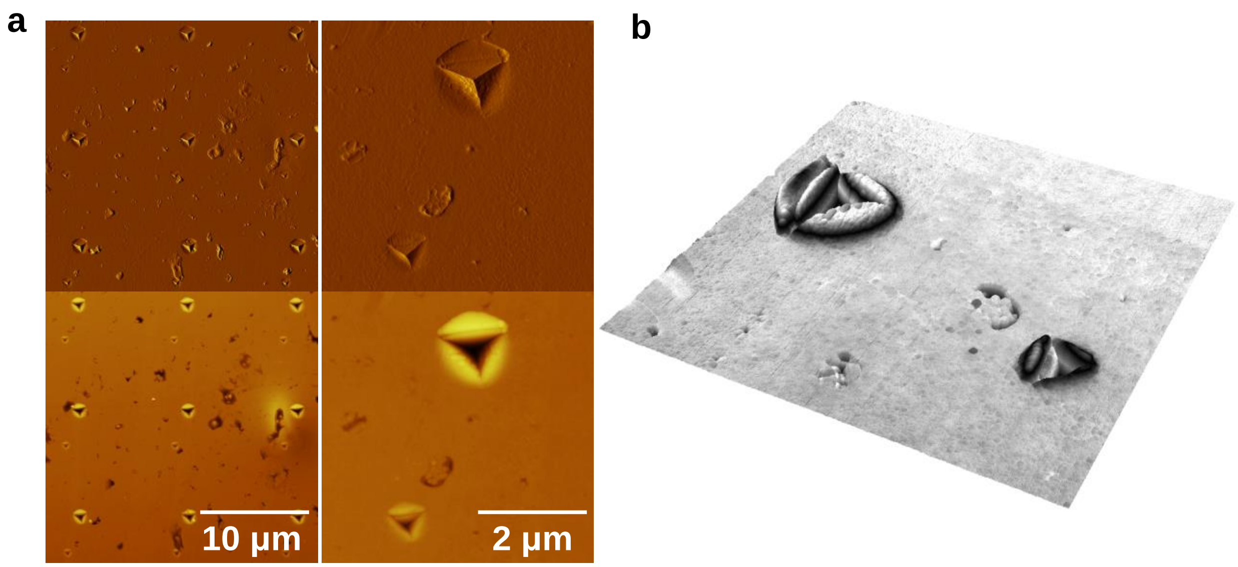

Supplementary Figure 15. Indentation imaged by AFM. Post-indentation AFM images of $(UPyU)_3TMP$. **a**, AFM images showing a pattern of indentations and **b**, a 3D image visualizing the indentation caused by loads of 300 µN (top left) and 150 µN (bottom right). The image shown in **b** was created by converting two imprints shown in **a** in 3D using MountainsMap Premium software. Indentations were made prior to imaging using a cube corner diamond DNSIP cantilever by AFM in force spectroscopy mode. After indentation a NanoWorld NCHR high resonance cantilever was mounted to record the images.

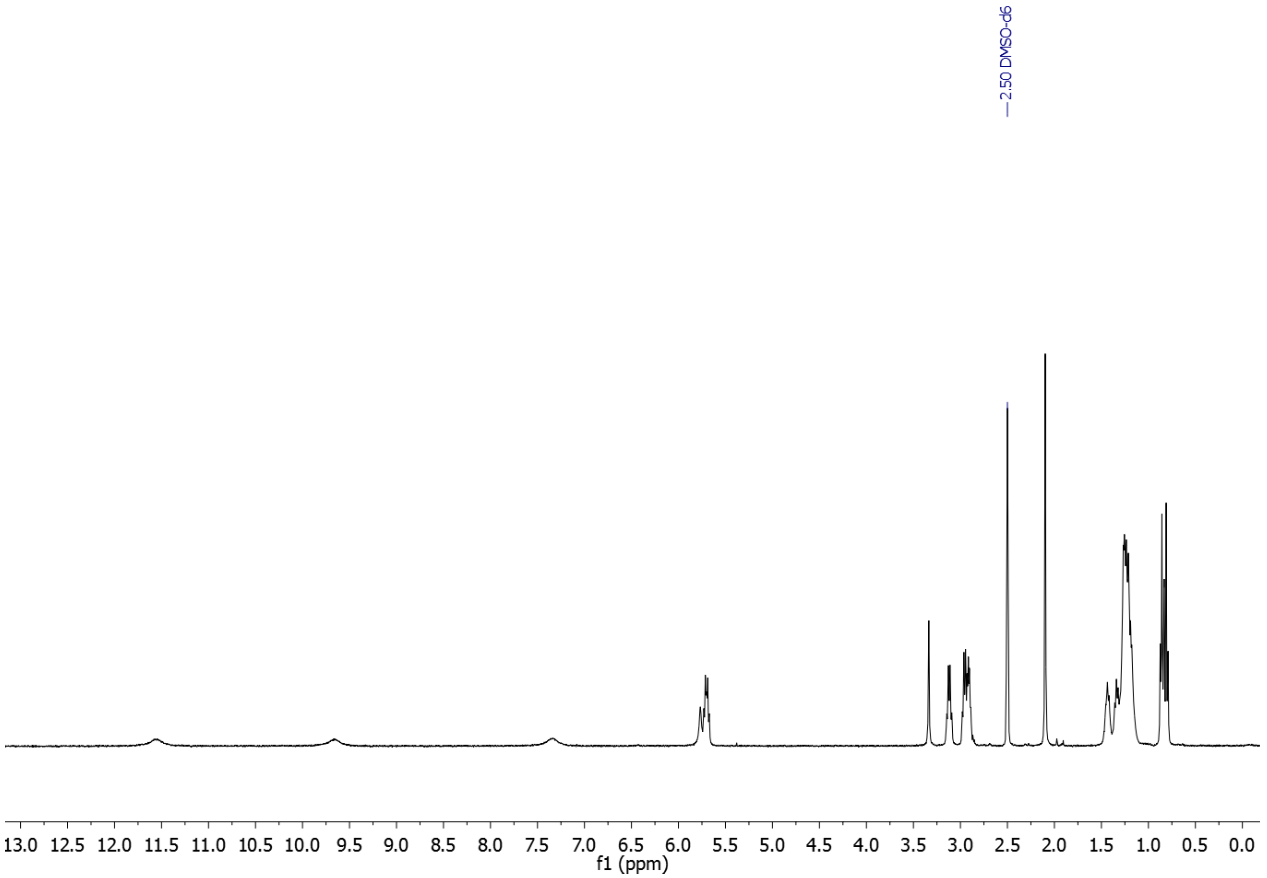

Supplementary Figure 16. ^{1}H-NMR spectrum of UPy-C6-U-EH. ^{1}H NMR (DMSO-*d*6, 360 MHz) δ 11.56 (s, 1H), 9.66 (s, 1H), 7.34 (s, 1H), 5.77 (s, 1H), 5.71 (q, 2H), 3.13 (q, 2H), 2.95 (dq, 4H), 2.10 (s, 3H), 1.44-1.25 (bm, 17H), 0.81 (dt, 6H).

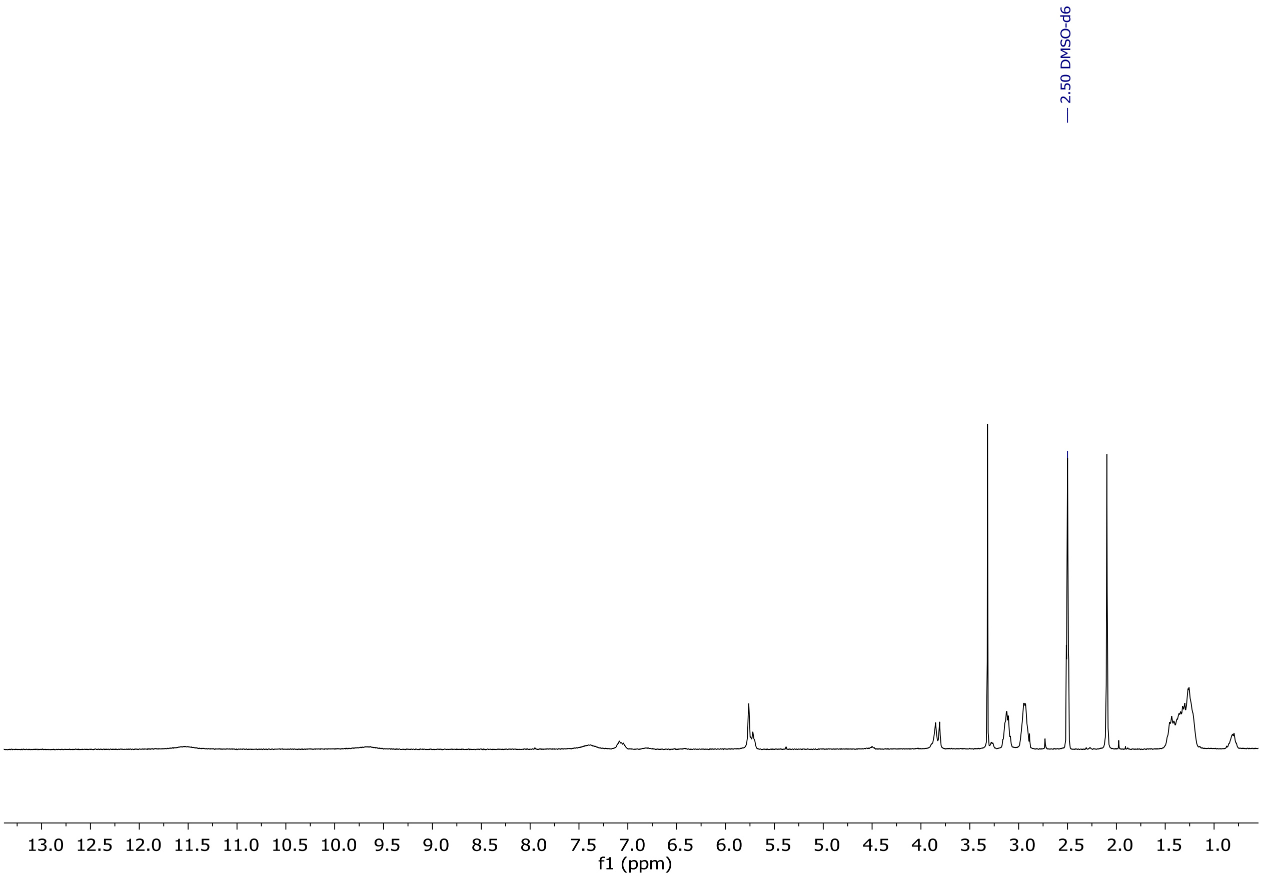

Supplementary Figure 17 ^{1}H-NMR spectrum of (UPyU)$_3$TMP. ^{1}H NMR (DMSO-*d*6, 360 MHz) δ = 11.53 (s, 3H), 9.67 (s, 3H), 7.39 (s, 3H), 7.09 (s, 3H), 5.76 (s, 3H), 3.85 (ds, 6H), 3.12 (t, 6H), 2.93 (s, 6H), 2.10 (s, 9H), 1.43 - 1.27 (bm, 26H), 0.8 (s, 3H).
